# Bone Mineralization in Celiac Disease

**DOI:** 10.1155/2012/198025

**Published:** 2012-04-18

**Authors:** Tiziana Larussa, Evelina Suraci, Immacolata Nazionale, Ludovico Abenavoli, Maria Imeneo, Francesco Luzza

**Affiliations:** Department of Health Science, University of Catanzaro “Magna Graecia”, University Campus of Germaneto, Viale Europa, 88100 Catanzaro, Italy

## Abstract

Evidence indicates a well-established relationship between low bone mineral density (BMD) and celiac disease (CD), but data on the pathogenesis of bone derangement in this setting are still inconclusive. In patients with symptomatic CD, low BMD appears to be directly related to the intestinal malabsorption. Adherence to a strict gluten-free diet (GFD) will reverse the histological changes in the intestine and also the biochemical evidence of calcium malabsorption, resulting in rapid increase of BMD. Nevertheless, GFD improves BMD but does not normalize it in all patients, even after the recovery of intestinal mucosa. Other mechanisms of bone injury than calcium and vitamin D malabsorption are thought to be involved, such as proinflammatory cytokines, parathyroid function abnormalities, and misbalanced bone remodeling factors, most of all represented by the receptor activator of nuclear factor B/receptor activator of nuclear factor B-ligand/osteoprotegerin system. By means of dual-energy X-ray absorptiometry (DXA), it is now rapid and easy to obtain semiquantitative values of BMD. However, the question is still open about who and when submit to DXA evaluation in CD, in order to estimate risk of fractures. Furthermore, additional information on the role of nutritional supplements and alternative therapies is needed.

## 1. Epidemiology of Bone Involvement in CD

Since 1980s, the most widely used tool in osteoporosis detection, treatment, and follow-up has been dual-energy X-ray absorptiometry (DXA) which showed a strong correlation between detection of bone mineral density (BMD) and fracture risk. Other procedures used to assess BMD include dual-photon absorptiometry (DPA), quantitative computed tomography (QTC), and ultrasound [1]. World Health Organization criteria for osteopenia and osteoporosis are defined by means of BMD as currently assessed by DXA indicating, respectively, a *T* score between −1 and −2.5 and ≤2.5. Both these conditions consist of a quantitative and qualitative alteration in the arrangement of bone tissue with a consequent increase in bone fragility and susceptibility to fracture [2].

Several studies evaluated bone status in celiac disease (CD), both at diagnosis and after gluten-free diet (GFD), and to date, it has been recognized that bone involvement may be a frequent finding during CD. Nevertheless, studies focusing on the prevalence of bone derangement in celiac patients are still inconclusive since both old and recent findings fall in a wide range ([3, 11], see Table 1).

On the other hand, the prevalence of CD in idiopathic osteoporotic patients has been investigated in many studies, but controversy still does exist about the value of screening for CD in this setting. Duerksen and Leslie [12] observed that adult women who were positive for antibody testing for CD had lower BMD than the seronegative control group. Stenson et al. [13] reviewed screening results for CD in osteoporotic patients and found a 3.4% incidence of CD compared to 0.2% among general population in subjects without gastrointestinal symptoms. In 1992, Lindh et al. [14] screened 92 patients with osteoporosis for CD showing that 11 (12%) had elevated levels of serum IgA antibodies to gliadin, while only three of them displayed CD-related intestinal lesions. More recently, among 135 patients with low BMD evaluated by Karakan et al. [15], 13 (9.6%) displayed positivity for IgA antiendomysial antibodies, but histological examination of intestinal mucosa was normal in all of these patients. Also, Mather et al. [16] did not detect an increased prevalence of CD among 100 consecutive patients referred for evaluation of low BMD. Indeed, despite a high rate of weakly positive IgA antiendomysial antibodies tests (7.3%), none of these subjects showed histopathological features of CD at the small bowel biopsy. Data are summarized in Table 2. Therefore, a screening strategy for CD in subjects with reduced BMD does not seem to have a major role in order to identify a secondary cause of bone impairment. Furthermore, clinicians should take into account the cost of CD serology tests that precludes their large-scale use. Maybe, screening tests for CD in idiopathic osteoporosis should be addressed to selected patients with no evidence of well-established risk factors for osteoporosis (i.e., younger, premenopausal, male gender patients).

## 2. Pathophysiology of Bone Metabolism in CD

### 2.1. Bone Metabolism in Adults

Individual's gender, constitution, and age as well as variations in endocrine systems associated with factors such as menopause and presence of comorbidities can all interact with lifestyle factors, including smoking, lack of exercise, and low dietary calcium intake to determine the onset of osteoporosis [17].

Bone is a dynamic tissue continuously renewed in a process called bone remodeling which is highly regulated by means of a complicated mechanism. However, the peculiar molecular pathways that control its initiation, progression, and cessation remain poorly understood. A leading role relies on two types of cells: osteoclasts, which are differentiated monocyte-derived cells involved in the removal of bone matrix, and osteoblasts, which derive from mesenchymal stem cells and are capable to form new bone. In the third decade of life, the process of bone resorption begins to exceed bone formation, and this fact leads to a progressive bone loss [18].

Nutrition plays an important role in bone homeostasis, providing the necessary substrates for the metabolic functions of bone tissue, most at all vitamin D and minerals. Vitamin D regulates intestinal calcium absorption by stimulating the formation of specific proteins that transport calcium through enterocytes, called calbindin and calcium-binding proteins. There are two forms of vitamin D: D3 (cholecalciferol) and D2 (ergocalciferol). Both forms are biologically activated in humans by hydroxylation first in the liver, to form 25-hydroxyvitamin D (25-[OH]D), and then in the kidneys, to form 1,25-dihydroxyvitamin D (1,25-[OH]_2_D). Even with low biological activity, 25-(OH)D is the main circulating form of vitamin D; therefore, blood 25-(OH)D concentrations are generally thought to reflect nutritional status regarding vitamin D. Furthermore, reduced calcium intake or malabsorption leads to increased parathyroid hormone (PTH) secretion which promotes bone turnover and cortical bone loss. PTH and 1,25-(OH)_2_D are linked in a series of coordinated activities to maintain normal serum calcium levels. When circulating calcium is reduced, the parathyroid glands increase the secretion of PTH, which in turn increases the circulating levels of 1,25-(OH)_2_D, by stimulating the renal hydroxylation of 25-(OH)D. This is the reason why increased 1,25-(OH)_2_D levels may be observed in CD [19].

#### 2.1.1. Malabsorption

The impact of nutrient malabsorption caused from untreated CD is well documented. In patients with symptomatic CD, the main cause of low BMD is related to the state of malabsorption. Impaired absorption of calcium during CD is thought to result principally from loss of villous in the proximal intestine, where calcium is most actively absorbed. Adherence to a strict GFD will reverse the histological damage in the intestinal mucosa and also the biochemical evidence of calcium malabsorption, as demonstrated with the use of strontium test by Molteni et al. [20]. However, vitamin D receptors are normally expressed in the duodenal mucosa of celiac patients, even in the presence of villous atrophy, suggesting that additional mechanisms other than calcium malabsorption due to villous atrophy are possibly involved in bone injury [21]. Pazianas et al. [22] showed a reduced fractional calcium absorption compared with controls in female patients on GFD from a mean duration of 4.7 years, notwithstanding variable degrees of improvement of intestinal mucosa. In this regard, it is not secondary to consider the role of the unabsorbed fatty acids in celiac patients. Indeed, intraluminal fats bind calcium in the intestinal lumen and may reduce dietary vitamin D absorption. Staun and Jarnum [23] showed a lack of calbindin and calcium-binding protein, the vitamin D-regulated proteins implicated in calcium uptake from the intestinal lumen, in the areas of damaged mucosa. 

#### 2.1.2. PTH and Hormone Disorders

It is well recognized that an excess of PTH can be associated with bone loss. Selby et al. [24] demonstrated a reduced BMD related to secondary hyperparathyroidism without vitamin D deficiency in patients on GFD. In a prospective study by Valdimarsson et al. [25], patients with initial secondary hyperparathyroidism displayed low BMD up to 3 years after GFD suggesting that different pathways in bone homeostasis of celiac patients are involved other than calcium malabsorption due to gluten-related damage of intestinal mucosa.

On the other hand, Lemieux et al. [26] performed a study on 17 treated celiac patients in order to assess the relationship between PTH levels, parathyroid function abnormalities, and bone loss. They confirmed a reduced BMD in all patients notwithstanding a 5.7-year mean period of GFD, but PTH values, although higher than in control group, were still in the normal range. Results regarding parathyroid functional studies were similar in both celiac and control group, excluding a residual secondary hyperparathyroidism in treated celiac patients.

Celiac males are also at greater risk of infertility and hypogonadism. In this case, CD patients are more likely to develop osteoporosis. On the other hand, hypogonadism in men may be often associated with hyperprolactinemia; thus, the occurrence of bone loss can be due to secondary increased levels of estrogens. Controversial opinions do exist about testosterone therapy in men for the prevention and treatment of osteoporosis. Nevertheless, it has been shown that men with osteoporosis and concomitant hypogonadism, as well as those with CD associated, may obtain beneficial effects from this treatment [17].

#### 2.1.3. Proinflammatory Cytokines

Recent studies showed that chronic release of proinflammatory cytokines, hormonal components, and other misbalanced bone remodeling factors can predispose celiac patients, either or not on GFD, to mineral metabolism derangement. Fornari et al. [27] found high levels of circulating IL-1*β* and IL-6 in untreated celiac patients and a reduction after GFD. In the same study, treatment produced an increase in IL-1 receptor antagonist levels which were normal at baseline evaluation, while serum levels of IL-6 negatively correlated with BMD. These findings suggest that these cytokines might have a role in the bone homeostasis during CD. In a review paper, Tilg et al. [28] pointed out the involvement of TNF-*α* and IFN-*γ* in bone remodeling, suggesting that their enhanced production and releasing during chronic inflammation is associated with increased bone loss.

Insights on the molecular mechanisms regulating osteoclast formation and activation progressed a lot in the past 15 years, with the identification of the receptor activator of nuclear factor kappa B/receptor activator of nuclear factor kappa B-ligand (RANK/RANKL) signaling system as well as the discovering of osteoprotegerin (OPG), a protein that appeared to protect from excessive bone reabsorption. Bone homeostasis is reached by a dynamic balance between bone reabsorbing activity performed by RANKL and the effects of its natural decoy receptor OPG. Fiore et al. [29] demonstrated that OPG/RANKL ratio was significantly lower in celiac patients with recovery of intestinal mucosa than in healthy controls and that positively correlated with low BMD.

In a brief paper by Riches et al. [30], autoantibodies against OPG were detected in a man with celiac disease who presented with severe osteoporosis and high bone turnover. Authors demonstrated that these autoantibodies had the potential to block the inhibitory effect of OPG on RANKL, and this led to the hypothesis that they may play a role in the development of bone derangement. In the same paper, circulating autoantibodies against OPG were detected in three among 15 additional patients with CD and low BMD, while there was no evidence of them in serum specimens from 10 healthy controls and 14 patients with autoimmune hypothyroidism. If these CD patients were or were not on GFD was not indicated by the authors, and data on duodenal mucosa histology were not provided. If circulating autoantibodies against OPG play a role in the pathogenesis of bone derangement in patients with CD, and to what extent, remains to be established. Indeed, in a more recent study, no evidence of these antibodies was found in the serum of 30 celiac patients on GFD independent of BMD, duodenal histology, and HLA status [31].

#### 2.1.4. Diet

Naturally gluten-free products are often low in B vitamins, calcium, vitamin D, iron, zinc, magnesium, and fiber. On the other hand, few gluten-free products are enriched or fortified. Bardella et al. [32], in evaluating nutritional status and body composition of 71 adult celiac patients who adhered to GFD and displayed normal histological findings at repeat duodenal biopsy, demonstrated that BMD of adulthood diagnosed patients was significantly lower than controls. In this group, female patients showed a nutritional unbalanced diet with higher percentage of energy as fat and lower percentage of energy as carbohydrates, thus concluding that dietary advice in celiac patients other than gluten withdrawal seems to be necessary in terms of the choice and composition of foods, in order to prevent complications due to malnutrition. A dietician must be part of the health care team to monitor the patient's nutritional status and compliance on a balanced diet.

Kinsey et al. [33] described a mean daily calcium intake below the recommended 1500 mg per day and an impaired intake of vitamin D in 92% and 62%, respectively, among 106 celiac patients on GFD who participated in a dietary survey.

The real impact of vitamin D deficiency in CD is not well established at date. While Bai et al. [6] observed amelioration of BMD in celiac patients receiving calcium and vitamin D supplements compared to GFD only, Ciacci et al. [34] did not find any additional benefit from such supplementation. In a randomized prospective study, Caraceni et al. [35] evaluated BMD at baseline and after 1 year GFD in two groups of celiac patients, one receiving vitamin D orally and one who did not. No significant differences in BMD levels were found in either groups, thus suggesting a non major role for vitamin D deficiency in this setting.

### 2.2. Bone Metabolism in Children

During childhood and early adulthood, bone formation generally equals bone resorption, favouring the maintenance of a constant bone mass. The most rapid gain in bone mass occurs during adolescence with bone mineral accretion accelerating dramatically along with the onset of puberty, while a less consistent fraction is subsequently acquired between the ages of 20 and 30 years. If normal peak bone mass is not achieved during those critical early years, subject is at higher risk for developing osteoporosis; thus, the amount of bone accrued during the pediatric years is an important predictor of an individual's future resistance to fractures [36].

#### 2.2.1. Malabsorption

During childhood, villous atrophy due to mucosal damage sustained by CD impairs intestinal absorption of nutrients, including the amount of calcium needed for bone accruement. Abnormal bone formation in children is an important problem for paediatricians because skeletal derangement consequences on growth are often of great importance as well as irreversible. Tau et al. [37] observed that 93% of children who started treatment before the age of 4 years reached normal spine BMD values, compared to 50% of those who were older at the time of diagnosis and gluten withdrawal. So, it can be concluded that individuals with short-term exposure to gluten are more likely to normalize their bone alterations, as a result of an optimal restoration of intestinal mucosal damage. Nevertheless, celiac children on diet for less than 12 months displayed significantly lower BMD than those on diet for more than 24 months [38].

#### 2.2.2. PTH and Hormonal Disorders

Recent data demonstrated that bone remodeling is under endocrine control; thus, a peculiar interest for pediatricians is represented by the role of hormones and specific growth factors in the mediation of bone turnover. Secondary hyperparathyroidism could be found also in celiac children in response to hypocalcemia. In a study by Zanchi et al. [39], PTH serum concentration was higher in children with CD than in control subjects but normalized after six months GFD. Conversely, normal serum PTH levels were found in celiac children at the time of diagnosis and during the followup period by Barera et al. [40] suggesting that an increased availability of calcium in younger patients than adults may prevent hypocalcemia and secondary hyperparathyroidism. During infancy and adolescence, GH stimulates growth and sexual development as well as increasing muscle mass and the formation of bone tissue. GH deficiency was found in children with CD referred for short stature and showing no catch-up growth after 1-year GFD [41]. Since growth hormone (GH) secretion may significantly affect BMD in children, DXA scans in this setting should be evaluated with caution to avoid the risk of overestimating bone damage before treating GH deficiency. In this situation, replacement of GH therapy should be considered given the great impact of such a deficiency on the growth process. Insulin growth factor 1 (IGF-1) is essential for bone longitudinal growth; it plays a role in trabecular and cortical bone formation, and its relative deficiency may result in reduction in skeletal longitudinal growth. Federico et al. [42] evaluated IGF-1 and its binding proteins in 14 children with celiac disease, either before or after a 6-month gluten-free diet, and described a reduction of blood levels of IGF-1 and growth hormone-binding proteins during the active phase of CD which disappeared during the GFD. Also Jansson et al. [43] described a decrease of IGF-1 and its binding proteins in 54 celiac children who participated in a 4-week gluten challenge, and these findings independently correlated with weight change and small intestinal inflammation.

Another factor affecting bone remodeling in young celiac patients is the hormone leptin, the lack of which could be related to growth and puberty anomalies. Indeed, it is involved in a regulatory loop that appears to explain the protective effect of obesity on bone mass in humans. Leptinaemia levels were found to be low and to significantly increase after GFD in patients with severe intestinal atrophy [44].

#### 2.2.3. Proinflammatory Cytokines

Garrote et al. [45] studied the complicated cytokine network involved in the pathogenesis of CD in childhood and described a particular amount of IFN-*γ* in the intestinal mucosa along with an increased production of IL-15, IL-18, and IL-21 linked to gluten intake. Also, Mora [46] in a review paper article speculated that increased production of inflammatory cytokines may disrupt bone metabolism equilibrium in children and adolescents with CD. Studies on the relationship between increased pro-inflammatory cytokines and bone alteration in children are scanty. Nevertheless, available findings suggest that the inflammatory pathway is involved in the development of bone impairment in celiac children as it is in adulthood diagnosed patients.

#### 2.2.4. Diet

Monitoring dietary compliance is important to ensure appropriate bone mass accrual throughout childhood and puberty in CD patients. Adherence to a strict GFD worsens the already nutritionally unbalanced diet of adolescents, increasing elevated protein and lipid consumption despite a low carbohydrate intake [47]. Several dietary surveys observed an inadequate calcium intake among children and adolescents on GFD although the relationship between a given serum vitamin D levels and health outcomes such as peak bone mass and fracture risk in CD children is still unclear. Vitamin D deficiency may have affected bone matrix mineralization at diagnosis due to impaired mucosal absorption even though suboptimal vitamin D and K serum levels have been found in these patients even one year after GFD [48]. Blazina et al. [49] showed that in children and adolescent, who strictly adhered to GFD and did not display low BMD, calcium intake and vitamin D levels were below recommendations. Therefore, efforts should be made to ensure an adequate calcium intake and vitamin D supplementation in this setting.

**Figure 1:**
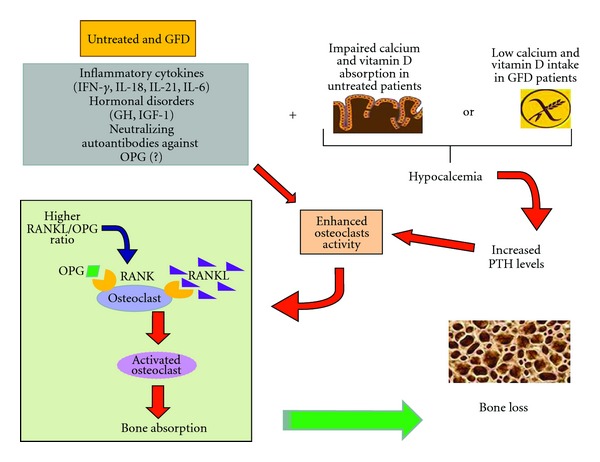
Mechanisms involved in the pathogenesis of bone derangement in celiac disease. GFD: gluten-free diet; IFN*γ*: interferon-gamma; IL: interleukin; IGF-1: insulin growth factor-1; GH: growth hormone; OPG: osteoprotegerin; RANK/RANKL: receptor activator of nuclear factor kappa B/receptor activator of nuclear factor kappa B-ligand.

Mechanisms involved in the pathogenesis of bone derangement in CD are reported in Figure 1.

## 3. Clinical Aspects of Low BMD in CD

### 3.1. Screening for Osteoporosis in Adults

Considering that an impaired bone mass is described in both symptomatic and asymptomatic CD patients, the question arises about which patients should undergo bone mass evaluation. Despite the high prevalence of bone demineralization in CD, there is still not a consensus about the timing to perform densitometric studies. In women, postmenopausal DXA is more sensitive for detecting osteoporosis, but it could lead to a delayed diagnosis in order to achieve a bone density gain with a proper treatment. At this regard, a screening DXA at diagnosis may detect an important bone involvement allowing an early management of the disease. However, Lewis and Scott [50] in the clinical application of these guidelines in a district general hospital found a low percentage of osteoporosis in newly diagnosed celiac women who underwent DXA scan. Furthermore, data show that CD-associated low BMD responds to GFD with gradual increase of bone mineralization. In particular, a five-year follow-up study by Kemppainen et al. [51] showed a significant improvement in BMD, mostly occurring in the first year from gluten withdrawal. These findings suggest that referring patients to DXA at diagnosis of CD may overestimate the bone involvement with the risk of overtreating patients who may actually benefit of GFD alone.

### 3.2. Screening for Osteoporosis in Children

According to some authors, the BMD screening question in CD patients must be addressed differently in childhood. Zanchi et al. [39] detected 18% osteopenia at DXA scan in 54 untreated children and demonstrated bone improvement after 6-month GFD, concluding that an expensive study of bone metabolism is not necessary in children with CD shortly exposed to gluten. On the other hand, recent data show a less-than-optimal peak bone mass value even after two-year GFD in children with CD while biochemical markers not performing as useful tools to assess BMD impairment [52]. Kalayci et al. [53] conclude that at least 4 years of GFD are required for a complete recovery of bone mineralization in some childhood patients and even suggest annually evaluation of BMD to clarify whether bone loss is completely recovered. Indeed, the main concern is that an altered bone development during childhood can affect final growth of the child. In this context, measurement of BMD should be included in the routine management of such children in order to implement appropriate treatment strategies and prevent long-term complications associated with poor bone health.

An additional point to discuss refers to the method for assessing bone health in childhood. Indeed, there is a debate whether DXA is an appropriate tool for studying BMD in children. Gafni and Baron [54] analyzed 34 children diagnosed with low BMD by means of DXA and found at least one error in interpretation in the 88% of the scans. The most frequent mistake was due to the use of *T*-score, that is, a standard deviation (SD) score referring to a comparison with young adults, instead of *z*-score, which indicates the difference in number of SDs between the mean BMD value of the individual and a group of people of the same sex and age. After correcting for these errors, 53% displayed normal BMD, and then half of the study population underwent a revision of their measurement. Therefore, physicians who engage DXA evaluations in children should be aware of these devices potentially leading to misdiagnosis.

### 3.3. Risk of Fracture

A special concern arises from the risk of fracture associated with bone demineralization in CD. Given that few studies addressed the actual fracture risk in this setting, the clinical impact of reduced BMD in CD is not well established. Furthermore, as assessed by Marshall et al. [55] in a meta-analysis of prospective cohort studies, the predictive value of DXA is not suitable enough to accurately identify subjects who will sustain fractures.

Sánchez et al. [56] evaluated the incidence and risk of peripheral fractures before and after diagnosis of CD in a cohort of 265 patients compared to a cohort of 530 age- and sex-matched controls. The CD group displayed significantly higher incidence rate and risk of peripheral fracture before diagnosis, particularly in men. The fracture risk was reduced after treatment and comparable results between the CD cohort and control group in both sexes. Jafri et al. [57] performed a population-based study in Olmsted County residents and investigated 83 celiac patients diagnosed between 1950 and 2002 and 166 gender and age-matched controls for fracture histories. A total of 39 (47%) cases had one or more fractures, with 40% occurring prior to their diagnosis date, compared to 45 (27%) controls. By means of a stratified proportional hazards model with comparable duration of follow-up in the two groups, the relative risk of having a fracture after the index date was greater in celiac patients than in their matched controls, concluding that not only fracture risk is elevated in CD, but this condition persists after the diagnosis. Thomason et al. [58] performed a large survey of patients with CD and found that 82 of 244 (35%) celiac patients and 53 of 161 (33%) age- and sex-matched healthy controls reported one or more fractures in their medical history. Accordingly, in a larger population-based case-control study which involved 1021 celiac patients, Vestergaard and Mosekilde [59] did not found a significant increase in fracture risk either before or after diagnosis of CD. Nevertheless, it must be taken into account the slight, even though not significant, increase in the risk registered in both studies, which might suggest a limitation of the study design in order to demonstrate a statistical significance rather than the absence of an association. Indeed, the sample size and the power of the study depend on the assumed fracture rate in the control population, which is different between studies. As highlighted by Walters and van Heel [60], femoral neck fractures have a population incidence of less than 1% in 65-years-old subjects but approaching 20% by the age of 90 years. However, in the study performed by Thomason et al., [58] only about one-third of individuals aged over 65 years. Therefore, approximately 400 cases and controls would be needed in a prospective study to detect a 50% increase in risk with a range from 20% to 30% and a 90% power. Findings are summarized in Table 3.

On the basis of current data, a correct conclusion might be that an increased fracture risk in CD cannot be excluded, but the clinical impact of this occurrence is relatively minor in celiac patients considered as a whole population.

## 4. Treatment of Bone Loss in CD

In children with CD, GFD is currently the first-choice therapy since it restores the intestinal malabsorption and therefore provides an improvement in bone mineralization process. This has been shown by Kavak et al. [61] in 28 childhood CD patients after one-year GFD who got mean BMD values comparable to those of healthy control subjects. Accordingly, Molteni et al. [62] demonstrated no significant differences in BMD between 22 patients treated from childhood and healthy sex- and age-matched controls, suggesting a long-term protective role for GFD when strictly followed since early age. Similar findings have been reported by Barera et al. [63] in a longitudinal study enrolling 20 patients (mean age: 10.12 ± 3.07 years) where DXA-assessed BMD at diagnosis has been found lower than in controls but became comparable one year after GFD. The beneficial impact of GFD on bone health has been confirmed by Cellier et al. [64] demonstrating that patients diagnosed in childhood and who had resumed normal diet in adolescence displayed bone complications in adult life. 

In CD patients diagnosed during adulthood, GFD is still considered to play a major role in bone health, even if it is not effective in completely reversing bone derangement by itself. McFarlane et al. [65] detected a significant gain in BMD after a 12-month GFD period in 21 newly diagnosed adult CD patients, even though there was still a lower BMD than in healthy controls, suggesting that there may be long-term impairment of bone mineralization in some otherwise healthy celiac patients who strictly adhere to a GFD. The effect of one-year GFD on bone health has been evaluated in the study by Sategna-Guidetti et al. [8] in 86 newly diagnosed adult patients where a significant improvement of lumbar spine and femoral neck mean BMD values has been demonstrated in 83.7% patients.

Few studies tested calcium and vitamin D supplementation in adult celiac patients, and current data did not provide evidence for additional benefits to GFD. In some special situations, such as osteoporosis detected in celiac postmenopausal women, it could be useful to begin a treatment with hormone replacement therapy or bisphosphonates (antiresorption agents). In addition, education on the importance of lifestyle changes, such as regular exercise, smoking cessation, and excessive alcohol intake, should be provided [66].

## Figures and Tables

**Table 1:** Prevalence of low bone mineral density in patients with celiac disease as assessed by dual-energy X-ray absorptiometry scan at spine.

Authors	Patients characteristics	Low BMD
*McFarlane et al., 1995 [3]	No. 65, on GFD	47%
Walters et al., 1995 [4]	No. 34, on GFD	38%
Valdimarsson et al., 1996 [5]	No. 63, untreated	38%
Bai et al., 1997 [6]	No. 25, untreated	72%
*Kemppainen et al., 1999 [7]	No. 77, on GFD and untreated	26%
Sategna-Guidetti et al., 2000 [8]	No. 86, untreated	66%
Meyer et al., 2001 [9]	No. 128, on GFD and untreated	72%
Motta et al., 2009 [10]	No. 31, on GFD	9%
Vilppula et al., 2011 [11]	No. 35, untreated	62%

BMD: bone mineral density; GFD: gluten-free diet.

*Established as osteoporosis.

**Table 2:** Prevalence of positive serology for celiac disease in patients with low bone mineral density.

Authors	Positive serology for celiac disease
Lindh et al., 1992 [14]	11 out of 92 (12%)
Mather et al., 2001 [16]	7 out of 96 (7.3%)
Stenson et al., 2005 [13]	12 out of 266 (4.5%)
Karakan et al., 2007 [15]	13 out of 135 (9.6%)

**Table 3:** Risk of fracture in celiac disease.

Authors	Comments
Marshall et al., 1996 [55]	DXA assessment does not accurately predict fracture risk
Vestergaard and Mosekilde, 2002 [59]	No differences before and after diagnosis of CD
Thomason et al., 2003 [58]	No difference in fracture history between CD and control patients
Jafri et al., 2008 [57]	Fracture risk is higher in CD patients, even on GFD
Sanchez et al., 2011 [56]	Fracture risk is comparable between CD and control patients

DXA: dual-energy X-ray absorptiometry; CD: celiac disease; GFD: gluten-free diet.
